# The evolution of animal Argonautes: evidence for the absence of antiviral AGO Argonautes in vertebrates

**DOI:** 10.1038/s41598-017-08043-5

**Published:** 2017-08-23

**Authors:** Niels Wynant, Dulce Santos, Jozef Vanden Broeck

**Affiliations:** Molecular Developmental Physiology and Signal Transduction, KU Leuven, Naamsestraat 59, P.O. Box 02465, B-3000 Leuven, Belgium

## Abstract

In addition to mediating regulation of endogenous gene expression, RNA interference (RNAi) in plants and invertebrates plays a crucial role in defense against viruses via virus-specific siRNAs. Different studies have demonstrated that the functional diversity of RNAi in animals is linked to the diversification of the Argonaute superfamily, central components of RISCs (RNA induced silencing complexes). The animal Argonaute superfamily is traditionally grouped into AGO and PIWI Argonautes. Yet, by performing phylogenetic analyses and determining the selective evolutionary pressure in the metazoan Argonaute superfamily, we provide evidence for the existence of three conserved Argonaute lineages between basal metazoans and protostomes, namely siRNA-class AGO, miRNA-class AGO and PIWI Argonautes. In addition, it shown that the siRNA-class AGO lineage is characterized by high rates of molecular evolution, suggesting a role in the arms race with viruses, while the miRNA-class AGOs display strong sequence conservation. Interestingly, we also demonstrate that vertebrates lack siRNA-class AGO proteins and that vertebrate AGOs display low rates of molecular evolution. In this way, we provide supportive evidence for the loss of the antiviral siRNA-class AGO group in vertebrates and discuss the consequence hereof on antiviral immunity and the use of RNAi as a loss of function tool in these animals.

## Introduction

A large portion of the RNA-species in Metazoans is not translated into proteins, but plays a role in controlling the cellular protein levels^1^. One of the most important and best-described groups of regulatory RNAs are the small RNAs, which trigger gene silencing through the RNA interference (RNAi) pathway^2^.

There are three major branches of small RNAs. Whereas genome encoded micro (mi)RNAs play a central role in the regulation of a multitude of cellular and physiological processes by induction of silencing of endogenous transcripts, short interfering (si)RNAs are derived from long exogenous dsRNA-fragments, typically produced during virus replication, and act as the defenders of the cell against invading viruses. A third group are the piwi-interacting (pi)RNAs, which help maintain the genome stability through silencing of selfish nucleic acids, like transposons^3–5^.

Notwithstanding the enormous complexity of RNAi, only three key protein components are well-conserved in eukaryotes, namely Argonaute proteins, RNA-dependent RNA-polymerases (RdRPs) and Dicer-like enzymes^6^. The diversity of RNAi appears to be linked to the presence of multiple paralogues of these RNAi-genes and a complex subset of accessory proteins. Argonaute proteins play a central role in binding to small RNAs and silencing of complementary transcripts in the cell by either destroying them or preventing their correct translation. Argonaute proteins found in animals are classified into three major groups, based on their interaction with specific small RNA species. Members of the first group bind to mi- and siRNAs, and are termed the AGO Argonautes, while the second group, the PIWI Argonautes, interact specifically with piRNAs. Nematode specific secondary (S)AGOs constitute the third branch of the Argonaute protein family^7, 8^. The latter includes Argonautes that interact specifically with newly synthesized (secondary) small RNAs, which are produced by RdRPs^9^.

The AGO group can be further functionally separated into AGOs specialized in mi- or siRNA-directed silencing. In insects, AGO1 is required for miRNA-directed silencing of endogenous transcripts, while AGO2 is needed for siRNA-based antiviral immunity^10^. Functional separation of these AGO proteins is further supported by the fact that *Drosophila* AGO2 was found to be under higher evolutionary pressure than AGO1, which is in accordance with its role in immunity. The situation in the nematode *Caenorhabditis elegans* is more complex, as 27 different Argonaute proteins are encoded in the genome of this worm, of which 19 are SAGO and 2 are PIWI proteins^9^. Still, the AGOs ALG1 and ALG2 are identified as the primary AGOs that mediate miRNA-directed regulation of endogenous gene expression, while RDE1 binds to exogenous (exo-)siRNAs to combat viral infections^11^ and ERGO1 interacts with endogenous (endo-)siRNAs to control expression of duplicating genes^9^.

Genome defence is presumed to be the ancestral function of RNAi in Eukaryotes^6^, and it remains a crucial antiviral pathway in plants, fungi and invertebrates^12–14^. Whether RNAi-based antiviral immunity also operates in vertebrates has been controversial for a long time^15–17^. Due to the development of alternative innate and adaptive immune systems, vertebrates can effectively combat viruses in the absence of RNAi^18^. Nevertheless, several studies using deep sequencing technologies identified low amounts of small virus-specific RNAs accumulating in mammals infected with viruses^19, 20^. Furthermore, virus encoded suppressors of RNAi (VSRs) have been identified in mammalian viruses^20, 21^. These recent findings reopened the debate on the existence of antiviral RNAi in mammals, and, more broadly, in vertebrates. In fact, it currently remains unclear to what extend viral-derived siRNAs contribute to the antiviral immune response in these animals.

Since an enormous diversity of Argonaute proteins is found in animals, with a clear functional specialization in the regulation of endogenous or exogenous transcripts, studying the phylogenetic relationship of Argonaute proteins can help understand the function of RNAi in different animal phyla. Phylogenetic analyses of the eukaryotic Argonautes superfamily have led to the identification of the AGO, SAGO and PIWI families^8^. Yet, the evolution of the Argonaute superfamily within animals, and in particular the evolution of the AGO subgroup in animals, has not yet been studied in detail.

Based on the principle of parsimony and the rate of molecular evolution, our analyses demonstrate, for the first time, that there exist two conserved AGO lineages between basal metazoans (sponges and cnidarians) and higher invertebrates, such as arthropods and nematodes, while vertebrates only maintained one type of AGO proteins.

## Results

### Identification of three conserved Argonaute lineages in metazoans

The mode of action of Argonaute AGO proteins in *C. elegans*, *D. melanogaster* and *Homo sapiens* has been well studied^9, 22, 23^, but the evolution of the AGO family in animals remains largely undetermined. Therefore, we constructed a ML phylogenetic tree using Argonaute proteins found in different species of nematodes, arthropods and chordates, as well as of flatworms and more primitive metazoans like poriferans (sponges) and cnidarians (jellyfish, corals and sea anemones).

**Figure 1:**
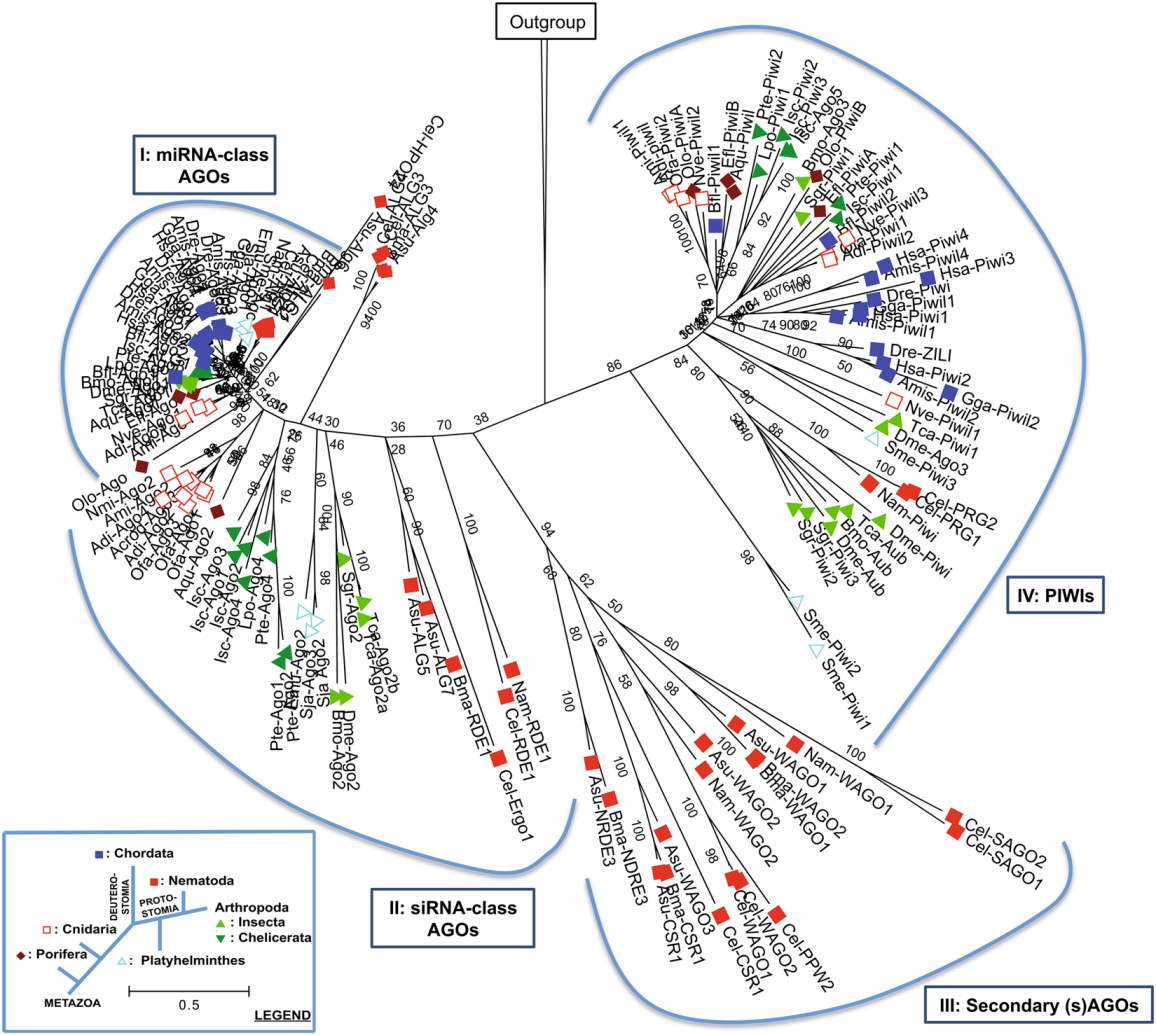
Rooted maximum likelihood phylogenetic tree (100 bootstraps) using the most conserved region between Argonaute superfamily members belonging to Porifera (: *Amphimedon queenslandica*; Aqu, *Ephydatia fluviatillis*; Efl, *Oscarella lobularis*; Olo), Cnidaria (: *Acropora digitifera*;﻿ Adi, *Acropora millepora*; Ami, *Orbicella faveolata*; Ofa, *Nemastella vectensis*; Nve), Chordata (: *Homo sapiens*; Hsa, *Alligator mississippiensis*;﻿ Ami, *Gallus gallus*; Gga, *Danio rerio*; Dre, *Brachiostoma floridae*; Bfl), Nematoda (: *Caenorhabditis elegans*; Cel, *Ascaris suum*; Asu, *Brugia malayi*; Bma, *Necator americanus*; Nam), Arthropoda (Insecta: : *Drosophila melanogaster*; Dme, *Bombyx mori*; Bmo, *Tribolium castaneum*; Tca, *Schistocerca gregaria*﻿; Sgr, and Chelicerata: : *Ixodes scapularis*; Isc, *Parasteatoda tepidariorum*; Pte, *Limulus polyp﻿hemus*; Lpo) and Platyhelminthes (: *Echinococcus multilocularis*; Emu, *Schistosoma japonicum*; Sja, *Schmistea mediterranea*; Sme). Detailed information on the sequences used is presented in Suppl. Table S2. The Argonaute superfamily can be dissected into four distinct groups: (I) miRNA-class AGOs, (II) siRNA-class AGOs, (III) Secondary (s)AGOs and (IV) PIWI Argonautes.

**Figure 2:**
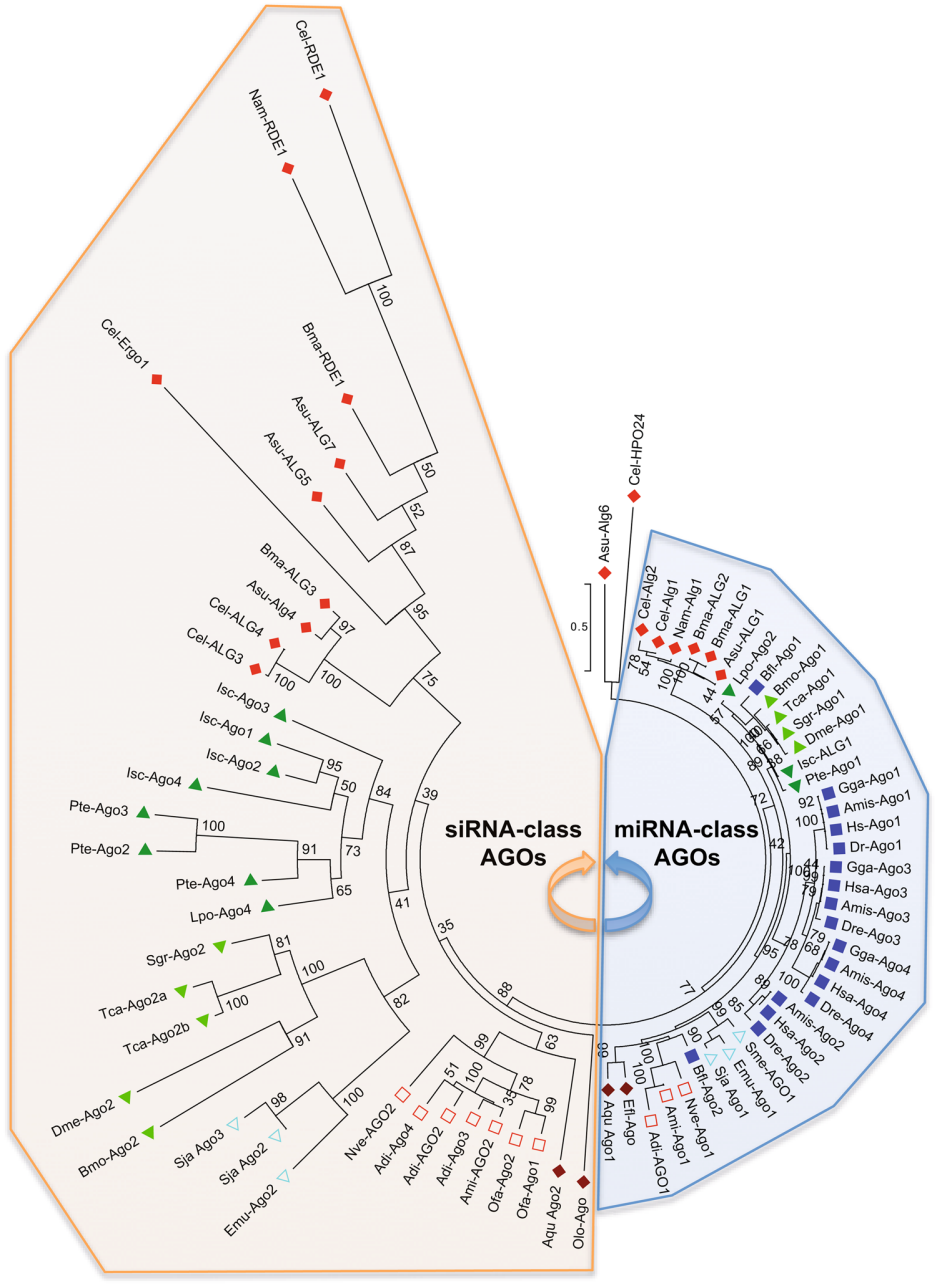
Unrooted maximum likelihood phylogenetic tree (100 bootstraps) using the AGO Argonaute members belonging to Porifera (: *Amphimedon queenslandica*; Aqu, *Ephydatia fluviatillis*; Efl, *Oscarella lobularis*; Olo), Cnidaria (: *Acropora digitifera*; Adi, *Acropora millepora*; Ami; *Orbicella faveolata*; Ofa, *Nemastella vectensis*; Nve), Chordata (: *Homo sapiens*; Hsa, *Alligator mississippiensis*, Ami; *Gallus gallus*; Gga; *Danio rerio*; Dre, *Brachiostoma floridae*; Bfl), Nematoda (: *Caenorhabditis elegans*; Cel, *Ascaris suum*; Asu, *Brugia malayi*; Bma, *Necator americanus*; Nam), Arthropoda (Insecta: : *Drosophila melanogaster*; Dme, *Bombyx mori*; Bmo, *Tribolium castaneum*; Tca, *Schistocerca gregaria*, Sgr﻿; and Chelicerata: : *Ixodes scapularis*; Isc, *Parasteatoda tepidariorum*; Pte, *Limulus polyphemus*; Lpo) and Platyhelminthes (: *Echinococcus multilocularis*; Emu, *Schistosoma japonicum*; Sja, *Schmistea mediterranea*; Sme). Detailed information on the sequences used is presented in Suppl. Table S2. The AGO Argonaute family can be dissected into two distinct groups: (I) miRNA-class AGOs and (II) siRNA-class AGOs.

Besides the PIWI and SAGO clades, two groups of AGO proteins can be distinguished (Fig. 1). (I) the first AGO group forms a separate monophyletic group containing sequences found in all groups investigated (Figs 1 and 2). Interestingly, within this group we find all the chordate AGO proteins, as well as the miRNA-interacting AGOs of *D. melanogaster* (Dme-AGO1) and *C. elegans* (Cel-ALG1 and 2). These fly and nematode AGOs were experimentally shown to mediate silencing of endogenous transcripts, but not antiviral immunity^9, 10^. In fact, studies have demonstrated that also AGO1 orthologues of locusts^24^ and human AGOs^25^ play a role in miRNA-directed endogenous gene regulation. Therefore, this clade appears to contain miRNA-interacting AGO Argonautes and is for this reason termed the miRNA-class AGO lineage. In addition, the studied poriferan, cnidarian and flatworm species also encode AGO proteins that cluster within this group, which is supported by a high bootstrap value (Figs 1 and 2). (II) The second AGO group does not form a monophyletic group when all Argonautes (AGOs, SAGOs and PIWIs) are included in the dataset (Fig. 1). Yet, when focusing specifically on the AGO subfamily, the existence of two distinct families is supported by high confidence (Fig. 2). This second group contains the Ecdysozoan (n﻿ematodes and arthropo﻿ds) AGOs that mediate siRNA-directed antiviral immunity, as experimentally demonstrated for Cel-RDE1 of *C. elegans* ^11^, Dme-AGO2 of *D. melanogaster* ^11^ and Isc-AGO2-4 of *Ixodes scapularis* ^26^. For this reason, we termed this group the siRNA-class AGOs. In agreement with a role in antiviral defense, the length of the branches, which represent the relative amount of amino acid substitutions, is clearly longer in this group than in the miRNA-class AGO clade (Figs 1 and 2). Amino acid sequence alignment of miRNA-class AGOs of *C. elegans* (Ce-ALG1), *D. melanogaster* (Dme-AGO1) and *H. sapiens* (Hsa-AGO2) revealed ≥60% sequence identity in the entire protein and >80% sequence identity in their PIWI domain. In contrast, amino acid sequence identity between siRNA-class AGOs of *C. elegans* (Cel-RDE-1 and Cel-ERGO-1) and *D. melanogaster* (Dme-AGO2) is less than 15% and even AGO2 proteins belonging to different insect species have very low sequence identity. For example, *D. melanogaster* and *B. mori* AGO2 possess only 23% sequence identity, while their housekeeping AGO1 counterparts share 81% sequence identity. Also the studied Cnidarian AGO1 proteins, which group in the miRNA class AGO clade, share much higher sequence similarity than their AGO2 counterparts, 75% sequence identity between AGO1s versus 18% between AGO2s of *Acropora millepora* and *Nemastella vectensis* (Table 1). It is fascinating that also the most primitive metazoan phyla possess two different AGO-type with clear differences in the rate of molecular evolution. While a direct role in antiviral immunity for the cnidarian and sponge AGO2 Argonautes has not yet been demonstrated, it seems reasonable to assume that the most recent common ancestor of all animals possessed, at least, two types of AGOs, an ancestral siRNA-class and miRNA-class AGO. (III) Nematodes encode an additional Argonaute clade, termed the SAGOs. SAGO proteins were found in all studied nematode species, but were absent in the other animal groups under investigation. Finally, (IV) PIWI proteins were found in all studied animal phyla and cluster as a monophyletic group. Nevertheless, *piwi* genes were absent in the available genome sequence information of the nematodes *Ascaris suum* and *Brugia malayi* and the flatworms *S* *chistosoma japonicum* and *Schmidtea mediterranea*. Making a phylogenetic tree with only the PIWI Argonautes as query resulted in the observation of two well-supported monophyletic subgroups (Supplementary Fig. S1). ﻿Both groups contain members of poriferan, cnidarian, flatworm, arthropod and vertebrate species, suggestion that also ﻿subfunctionalization of the PIWI Argonaute subgroups accurred ea﻿rly in animal evolution.

**Table 1:** Sequence identity and similarity between different metazoan miRNA-class AGOs (upper table) and siRNA-class AGOs (lower table).

Identity-Similarity (%)	Hsa-Ago2	Amis-Ago2	Cel-ALG1	Asu-ALG1	Dme-Ago1	Bmo-Ago1	Ami-Ago1	Nve-Ago1	Aqu-Ago1
**Hsa-Ago2**	x								
**Ami-Ago2**	94–94	x							
**Cel-ALG1**	60–67	57–65	x						
**Asu-ALG1**	60–68	58–66	79–84	x					
**Dme-Ago1**	64–72	63–69	60–70	63–72	x				
**Bmo-Ago1**	69–77	67–74	61–71	64–72	81–85	x			
**Ami-Ago1**	67–78	65–76	55–65	56–65	58–67	63–72	x		
**Nve-Ago1**	66–77	64–75	54–66	55–66	58–68	63–73	75–84	x	
**Aqu-Ago1**	59–70	55–66	55–66	56–68	58–68	60–71	56–67	56–67	x
**Identity-Similarity (%)**	**Dme-Ago2**	**Bmo-Ago2**	**Cel-RDE1**	**Cel-ERGO1**	**Asu-ALG5**	**Asu-ALG7**	**Ami-Ago2**	**Nve-Ago2**	**Aqu-Ago2**
**Dme-Ago2**	x								
**Bmo-Ago2**	23–36	x							
**Cel-RDE1**	13–22	14–29	x						
**Cel-ERGO1**	14–27	14–26	13–26	x					
**Asu-ALG5**	18–29	20–34	19–35	19–36	x				
**Asu-ALG7**	18–29	20–34	20–35	19–33	41–56	x			
**Ami-Ago2**	9–13	10–18	10–18	8–15	9–16	12–20	x		
**Nve-Ago2**	21–33	22–34	15–30	14–26	21–33	20–31	18–23	x	
**Aqu-Ago2**	19–29	23–35	17–31	16–31	27–41	26–39	17–25	29–40	x

The miRNA-class AGO sequences used are of *Homo sapiens* (chordate, Hsa-Ago2), *Alligator mississippiensis* (chordate, Ami-Ago2), *Caenorhabditis elegans* (nematode, Cel-ALG1), *Ascaris suum* (nematode, Asu-ALG1), *Drosophila melanogaster* (insect, Dme-Ago1), *Bombyx mori* (insect, Bmo-Ago1), *Acropora millepora* (cnidarian, Ami-Ago1), *Nemastella vectensis* (cnidarian, Nve-Ago1), and *Amphimedon queenslandica* (sponge, Aqu-Ago1). The siRNA-class AGOs used are of *C. elegans* (nematode, Ce-RDE1 and Ce-ERGO1), *A. suum* (nematode, Asu-ALG5 and Asu-ALG7), D. melanogaster (insect, Dme-Ago2), *B. mori* (insect, Bmo-Ago2), *A. millepora* (cnidarian, Ami-Ago2), *N. vectensis* (cnidaria, Nve-Ago2) and *A.* *queenslandica* (sponge, Aqu-Ago2). In each cell, the first number corresponds to the percentage of sequence identity, while the second value stands for the percentage of sequence similarity.

Due to the highly expanded AGO-family in nematodes, there are two additional AGO orthologues for which homologues were not found in the other animal groups under investigation. The HPO-24-like sequences, present in *C. elegans* and *A. suum*, form a separate cluster closely related to the miRNA-class AGO clade (Figs 1 and 2). In addition, ALG3/4-like sequences cluster within a clade containing the siRNA-class Argonautes Cel-RDE1 and Cel-ERGO1 (Figs 1 and 2). Yet, Conine and coworkers demonstrated that the ALG3/4 play an important role in promoting the transcription of spermatogenesis genes of *C. elegans* ^22^. Therefore, it appears that these additional nematode AGO-species are a result of duplication and (sub)functionalization of *miRNA-class* and *siRNA-class ago* genes, respectively, in the nematode phyla.

Finally, it appears that all chordates lack siRNA-class AGOs, since none of the available genome sequence information of chordates in Genbank possess siRNA-class AGO sequences. This is exemplified by the fact that the available chordate sequence with highest homology (represented by the lowest E-value) to the *D. melanogaster* AGO2 protein is *B. floridae* Bfl-AGO2 (Supplementary Table S1), an Argonaute that clusters within the miRNA-class AGO group (Fig. 2). Yet, due to the limited amount of chordate sequence information available on Genbank, it remains possible that siRNA-class AGOs are present in other primitive chordate﻿ species.

### Elevated rates of molecular evolution in siRNA-class AGOs and PIWIs, but not in miRNA-class AGOs in insects

Immune proteins are under strong selective pressure, due to the host-pathogen arms race. Directional selective pressure can be identified by testing for an elevated rate of non-synonymous nucleotide substitutions (Ka) compared to synonymous substitutions (Ks)^27^.

**Figure 3:**
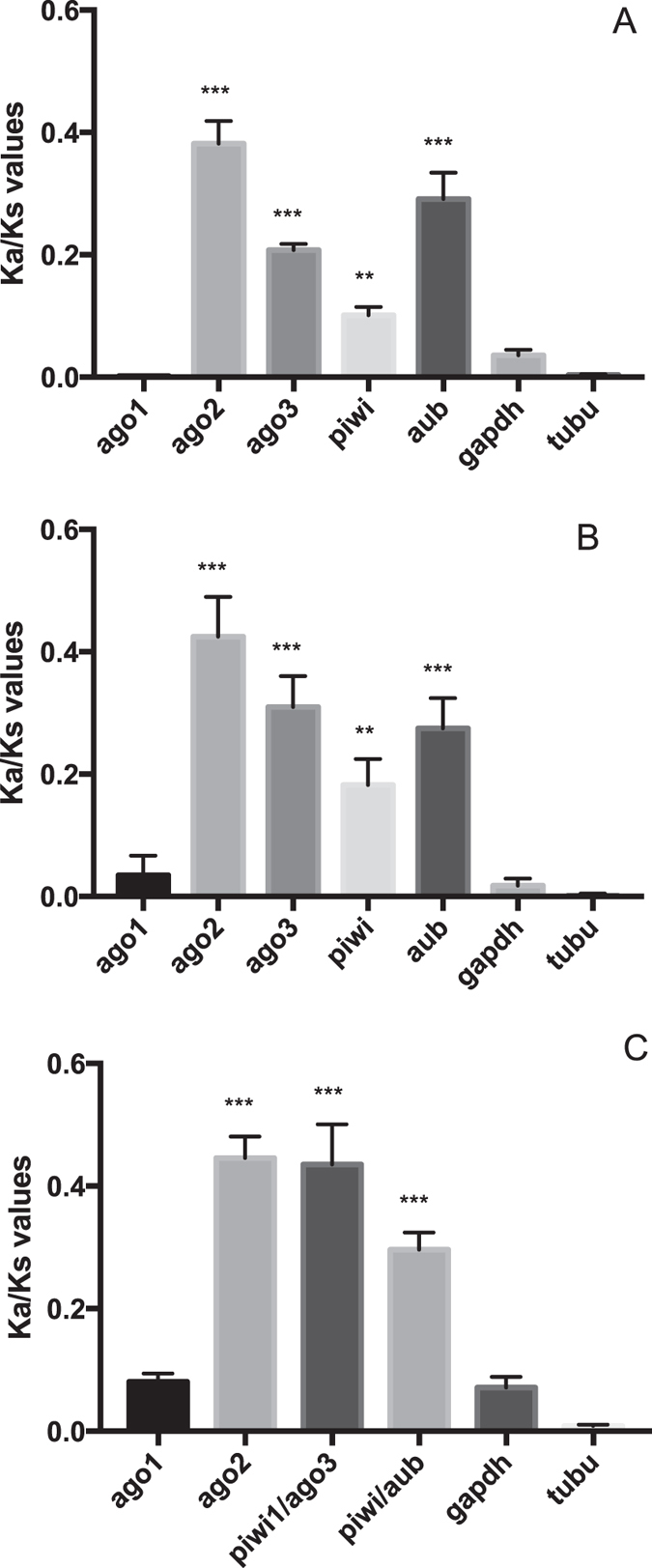
The evolutionary rate of *argonaute* sequences within the *Drosophila* genus, estimated by determining the Ka versus Ks substitutions by pairwise (Fig. 3A) and ML-tree based (Fig. 3B) comparison and using sequence information of *D*. *melanogaster*, *D. simulans*, *D*. *sechellia*, *D. yakuba* and *D. erecta*. Figure 3C: Rate of molecular evolution of *argonaute* sequences within the Insecta class. For this, sequence information of the *D. melanogaster*, *Bombyx mori*, *Tribolium castaneum* and *Schistocerca gregaria argonaute* coding regions was used as query. The bars represent the mean +/− SEM of individual Ka/Ks values per gene. Statistics (MWU-test) support difference with the housekeeping genes *glyceraldehyde 3-phosphate dehydrogenase* (gapdh) and *apha-tubulin1a* (tubu); **p < 0.01, ***p < 0,0001.

Utilizing tree-based and pairwise comparison for the calculation of Ka/Ks values of *Drosophila* RNAi genes, elevated rates of molecular evolution were identified in the *Drosophila sp. siRNA class-ago* (*ago2*) and *piwi* genes (*ago3, piwi* and *aub*), while not in the *miRNA-class ago* gene (*ago1*), *i.e*. in comparison to the studied housekeeping genes *alpha-tubulin1a* and *gapdh* (Fig. 3A and B). However, the ratio averaged over all sites is almost never >1, since positive selection is heterogeneous in nature, unlikely to affect all codons. Therefore, site-specific models in the Codeml package were used to allow the Ka/Ks value to vary among sites. Statistics for codon-specific selection were positive for *ago2*, *ago3* and *aub* (Table 2).

**Table 2:** Likelihood ratio tests (LRTs) for codon-specific evolutionary selection in *argonaute* sequences within *Drosophila sp*. and primates.

	lnL_M7_	lnL_M8_	2ΔlnL (LRT)	*p*-Value	Tree length
*Drosophilae*					
*ago1*	−4733.497	−4733.501	0.008	n.s.	0.232
*ago2*	−9056.855	−9046.185	21.341	**1.16 e-05**	1.008
*ago3*	−5278.170	−5275.845	4.650	**0.041**	0.710
*aub*	−5844.195	−5840.904	6.581	**0.019**	0.696
*piwi*	−5340.199	−5339.363	1.673	n.s.	0.593
**Primates**					
*ago1*	−4733.497	−4733.501	−0.008	n.s.	0.23791
*ago2*	−4167.933	−4167.937	−0.008	n.s.	0.31090
*ago3*	−3698.245	−3698.246	−0.001	n.s.	0.21646
*ago4*	−3601.807	−3601.809	−0.003	n.s.	0.08879
*piwil1*	−5197.149	−5197.130	0.03820	n.s.	0.45568
*piwil2*	−6167.099	−6167.045	0.10793	n.s.	0.49761
*piwil3*	−3672.889	−3668.723	8.33137	**0.0078**	0.64550
*piwil4*	−4812.804	−4812.653	−0.00002	n.s.	0.15997

The log-likelihood scores for model 7 (Neutral, LnL_M7_) and model 8 (Positive selection, LnL_M8_) were used to determine significance between these models. Therefore, the Chi-Square Distribution test (df = 2) was performed on the Likelihood ratio test (LRT) values. Sequences information of *D. melanogaster*, *D. simulans*, *D. sechellia*, *D. yakuba* and *D. erecta* was used to estimate codon specific evolutionary pressure within *Drosophila sp.* The primate species that were used for this analysis are *Homo sapiens*, *Pan troglodytes*, *Pan paniscus*, *Gorilla gorilla*, *Pongo abelli*, *Macaca mulatta* and *Callithrix jacchus*.

To test whether positive evolutionary selection in *ago2* and *piwi* sequences is specific for *Drosophila sp*. or whether this represents a more general characteristic of these genes in the entire insect class, the Ka/Ks values were estimated for *argonautes* of insect species belonging to different insect orders, namely of *D. melanogaster* (Diptera), *B. mori* (Lepidoptera), *T. castaneum* (Coleoptera) and *S. gregaria* (Orthoptera) (Fig. 3C). Elevated Ka/Ks values were detected for the tested *siRNA-class ago* and *piwi* genes, but not for *ago1*. Due to the high sequence divergence of these genes, pairwise comparison, as well as tests for codon-specific selection, could not be performed.

### Elevated rates of molecular evolution in PIWI proteins, but not in AGO proteins in vertebrates

**Figure 4:**
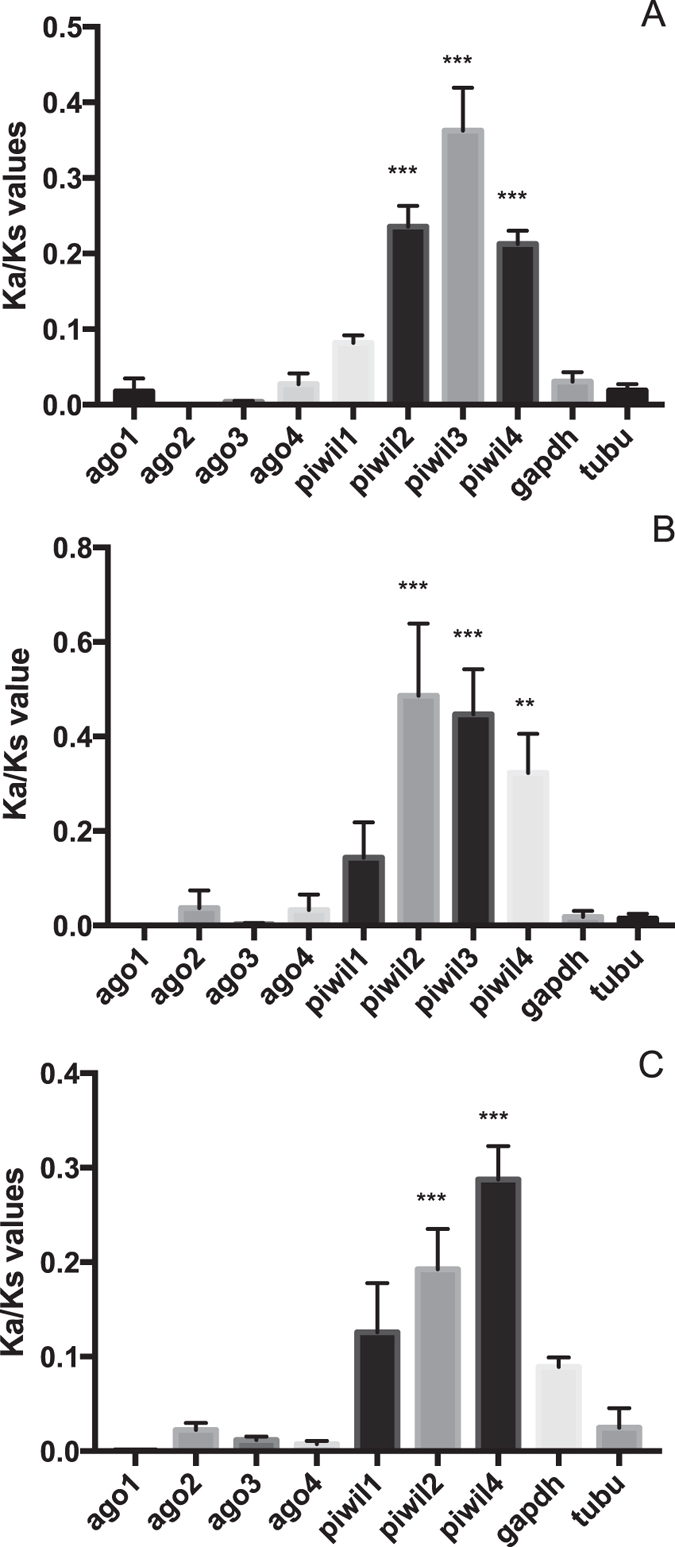
The evolutionary rate of *argonaute* sequences within the primates, determined by means of pairwise (Fig. 4A) and ML-tree based comparison (Fig. 4B). The nucleic acid sequence of Argonautes of *Homo sapiens*, *Pan troglodytes*, *Pan paniscus*, *Gorilla gorilla*, *Pongo abelli*, *Macaca mulatta* and *Callithrix jacchus* were used for Ka/Ks estimations. Figure 4C: Rate of molecular evolution of Argonautes within vertebrates. Ka/Ks values are estimated by means of ML-tree based comparison and by using sequence information of *H. sapiens*, *Mus musculus*, *Alligator mississippiensis*, *Gallus gallus* and *Danio rerio*. Noteworthy, Piwil4 is absent in the *G. gallus* and *D. rerio* genome sequence. The bars represent the mean +/− SEM of individual Ka/Ks values per gene. Statistics (MWU-test) support difference with the housekeeping genes *glyceraldehyde 3-phosphate dehydrogenase* (gapdh) and *apha-tubulin1a* (tubu); **p < 0.01, ***p < 0,0001.

To study directional selection in vertebrate *argonaute* genes, Ka/Ks values were estimated for *argonaute* genes of different primate species. Elevated Ka/Ks values were found in primate *piwi* genes (piwil2, 3 and 4), but not in their *ago* genes (Fig. 4A and B). In agreement with these observations, testing for site-specific directional selection was positive for *piwil2* and *piwil3* (Table 2), but negative for the *ago* genes. Furthermore, *piwil2* and *piwil4* are under elevated rates of selective pressure within vertebrates belonging to different classes (Fig. 4C). Similar as for the insect species with too high sequence divergence, pairwise comparison as well as tests for codon-specific selection could not be performed.

### Elevated rates of Ka/Ks substitutions in siRNA-class ago and sago genes, but not in miRNA-class ago and piwi genes in nematodes

**Figure 5:**
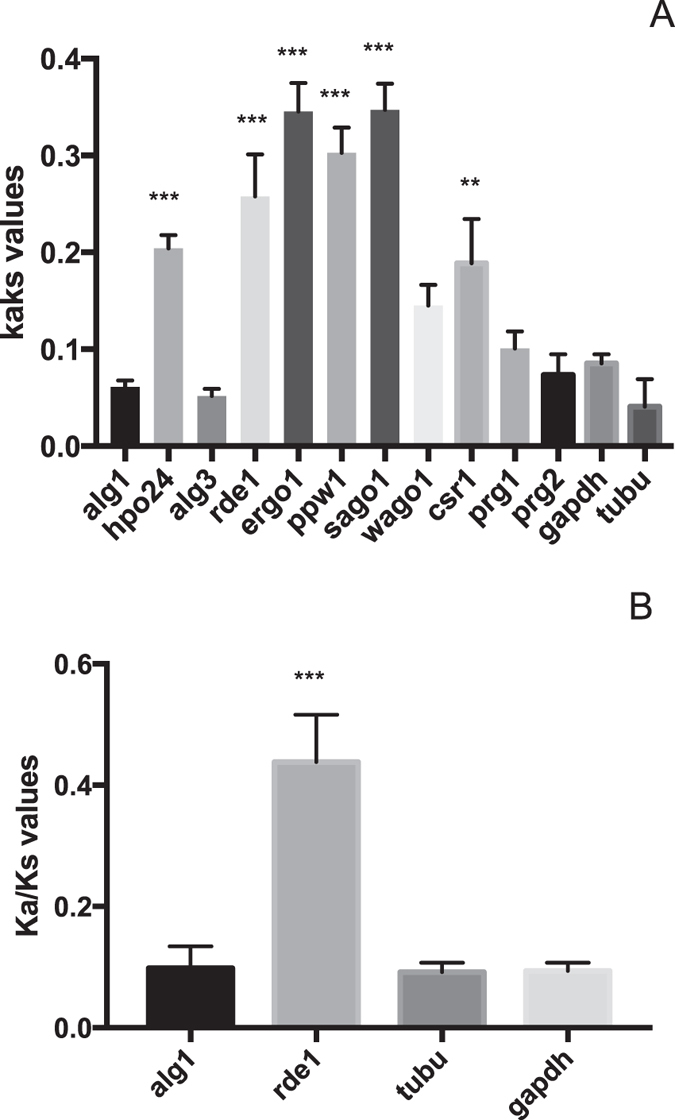
Figure 5A: Evolutionary rate of *argonaute* transcript sequences within the *Caenorhabditis* genus. Ka/Ks values are estimated by means of ML-tree based comparison and by using sequence information of *C. elegans*, *C. briggsae*, *C. remanei* and *C. brenneri*. Figure 5B: The evolutionary rate for Argonaute proteins in nematodes, determined by estimating the Ka/Ks values by means of ML-tree based comparison and by using sequence information of *C. elegans*, *Ascaris suum, Brugia malayi* and *Necator americanus*. Due to the enormous complexity and variation in the Argonaute AGO family present in nematodes, we could only find orthologs of the miRNA-class AGO *alg1* and the siRNA-class AGO *rde1* in all studied nematode species. The bars represent the mean +/− SEM of individual Ka/Ks values per gene. Statistics (MWU-test) support difference with the housekeeping genes *glyceraldehyde 3-phosphate dehydrogenase* (gapdh) and *apha-tubulin1a* (tubu); **p < 0.01, ***p < 0,0001.

Elevated rates of molecular evolution were also identified in *siRNA-class agos* (*rde1* and *ergo1*), *hpo24* and the *sago* genes of different *Caenorhabditis* species (Fig. 5A). Since *C. elegans* encodes 19 SAGO proteins, only four *sago* genes were selected for Ka/Ks estimations. Interestingly, *ppw1* and *sago1* displayed significantly higher Ka/Ks values than *wago1* and *csr1*. Noteworthy, most studied *Caenorhabditis* species appear to lack clear ALG-2 orthologues. Furthermore, in contrast to the situation in insects and vertebrates, we did not observe elevated Ka/Ks values for the nematode *piwi* genes (*prg1* and *prg2*). Unfortunately, we could not estimate the Ka/Ks values by means of pairwise comparison, nor study codon-specific directional selection, due to too low sequence similarity between the *siRNA-class ago* and *sago* genes of different *Caenorhabditis* species. Finally, the rate of molecular evolution between *argonautes* of species belonging to different nematode classes, namely of *C. elegans*, *A. suum*, *Necator americanus* and *B. malayi* was determined. Yet, we could only identify orthologues for ALG1 and RDE1 in the studied nematodes. The estimated Ka/Ks values for *rde1* were strongly elevated, but not these for the nematode *alg1* sequences (Fig. 5B).

## Discussion

This article describes the discovery of three conserved Argonaute groups in invertebrates, miRNA-class AGOs with a primary function in endogenous gene regulation, the antiviral siRNA-class AGOs that are under strong selective pressure and PIWI Argonautes that mediate piRNA-directed transposon silencing. The siRNA-class AGOs cluster in a well-supported monophyletic group when focusing on the AGO Argonautes (Fig. 2), but not when including all Argonautes (AGOs, SAGOs and PIWIs). A plausible explanation for this could be found in the high rate of sequence divergence of the siRNA-class AGO group, making it difficult to study their evolutionary relationship. Still, our data support the hypothesis that the genome of the most recent common ancestor of all animals encoded, at least, three different AGOs, an ancestral miRNA-class AGO, siRNA-class AGO and PIWI Argonaute. Furthermore, we demonstrate the exciting finding that vertebrates do not encode siRNA-class AGO Argonautes (Figs 1 and 2).

**Figure 6:**
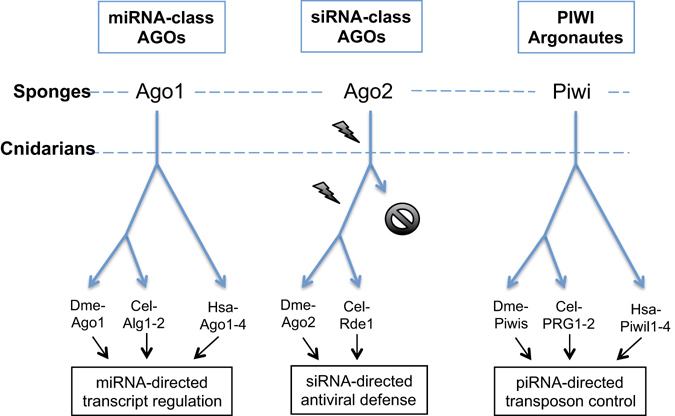
The evolution of the metazoan Argonaute superfamily. The miRNA-class AGO, siRNA-class AGO and PIWI Argonaute lineages are conserved between sponges, cnidarians, nematodes and insects, while chordates only encode miRNA-class AGOs and PIWIs (the absence of the siRNA-class AGOs in vertebrates is depicted by a black stop sign). Several studies have identified that the miRNA-class AGOs in the fruit fly (Dme-Ago1), *C. elegans* (Cel-ALG1-2) and humans (Hsa-Ago1-4) can interact with miRNAs and mediate regulation of endogenous transcripts (indicated with black arrows), the siRNA-class AGOs of flies (Dme-Ago2) and *C. elegans* (Cel-RDE-1) play a crucial role in the defense against viruses (black arrows), and finally, the PIWI Argonautes in flies (Dme-Piwis: Ago3, Piwi and Aubergene), *C. elegans* (Cel-PRG1-2) and humans (Hsa-Piwil1-4) mediate piRNA-directed control of transposons (also depicted by black arrows). The siRNA-class AGOs are characterized by elevated rates of molecular evolution (depicted by lightning signs).

The miRNA-class AGOs display strong sequence conservation (Table 1) and are not under elevated evolutionary pressure (Figs 3, 4 and 5), which is not in agreement with a direct role in antiviral immunity. This finding corresponds with previously published work, where it was shown that the analogous sequences in *D. melanogaster* (AGO1) and *C. elegans* (ALG1/2) function in the control of endogenous gene expression, but not in antiviral immunity^9, 11, 28^. Since vertebrates only encode miRNA-class AGOs (Figs﻿ 1, 2 and 4), our data support the absence of RNAi-based antiviral immunity in vertebrates. This is in accordance to the fact that deep sequencing of infected mammalian cells either failed to detect accumulation of virus-specific siRNAs or only identified extremely low levels^19^. Nevertheless, mammalian virus-encoded VSR have been identified, which can block RNAi efficiency in insect or plant systems^29–31^. One plausible explanation could be found in the fact that the majority of mammalian virus-encoded VSRs bind to dsRNA. While shielding the dsRNA might indeed block proper functioning of RNAi, it has also been reported that the VSRs have interferon-antagonistic properties^32–35^. This is an exciting finding bearing in mind that interferon is considered to be the first line of defense against viruses in mammals^36^. Interestingly, defence against invading nucleic acids is presumed to be the ancestral function of RNAi in eukaryotes^6^. Even more, the miRNAs of plants and animals appear to have evolved independently^6^. Therefore, it seems plausible to assume that the antiviral role of AGOs has been lost in vertebrates (Fig. 6).

Interestingly, in this paper, it is also demonstrated that several PIWI proteins in vertebrates are under evolutionary pressure (Fig. 4). Also for insects, elevated rates of evolutionary pressure were identified in PIWI proteins (Fig. 3), which is in accordance to the demonstrated antiviral role of the piRNA pathway in mosquitoes^37^. On the other hand, after deep sequencing of virus-infected *D. melanogaster* and silencing of the PIWI Argonaute proteins in these flies, Petit and colleagues (2016) concluded that the piRNA-pathway is not required for antiviral defense in the common fruit fly^38^. Nevertheless, we identified that the *Drosophila sp*. PIWI proteins are under elevated selective pressure (Fig. 4). This should not necessarily imply that the piRNA pathway plays a role in antiviral immunity in *Drosophila* flies, since other types of selfish nucleic acids, such as transposons, could be responsible for the identified evolutionary pressure in the PIWI proteins. In fact, it is well established that the PIWI pathway in *D. melanogaster* plays a crucial role in controlling retrotransposons^39^. Another interesting observation in the present study is the fact that selective pressure in the *Caenorhabditis* PIWI sequences is absent (Fig. 5A). Furthermore, *piwi* genes are absent in the genomes of *B. malayi* and *N. americanus* (Figs 1 and 2). In full agreement with this observation, Sarkies *et al*. (2015) sequenced small RNAs from multiple nematode species and observed that in the majority of studied species piRNAs are absent. In addition, they identified that RdRP-activity helps to compensate for the absence of piRNAs^40^. We also identified elevated rates of molecular evolution in the studied nematode SAGO proteins. SAGO Argonautes have only been identified in nematodes and are extremely diverse, with 19 SAGO genes in *C. elegans*. Interestingly, the rate of molecular evolution differs for the different studied SAGOs, with significantly higher rates of Ka versus Ks substitutions in *Caenorhabditis ppw1* and *sago1* than in *wago1* and *csr1* (Fig. 5). Remarkably, this corresponds well to the published results of Yigit and colleagues (2006), demonstrating that PPW1 and SAGO1 function primarily in long dsRNA-based RNAi, while CSR1 and WAGO1 act mainly in miRNA-based endogenous gene regulation^9^.

Besides its important role in the regulation of biological processes, RNAi is also a widely used tool for reverse genetics and holds potential for a vast array of application possibilities, such as the development of therapeutic agents and species-specific insecticides^5, 41, 42^. In insects and nematodes, long dsRNA, which is an important viral pathogen associated molecular pattern (PAMP), is used to activate the RNAi-pathway. In this way the antiviral siRNA-pathway is activated, which is supported by the observation that dsRNA-derived siRNAs are incorporated into the *D. melanogaster* AGO2 and the *C. elegans* RDE1 Argonautes^9, 28^. In order to purge a viral infection, the host must actively destroy as many viral transcripts as possible, with minimal off-target effects on endogenous transcripts. On the other hand, evidence from microarray studies indicated that a single miRNA can target hundreds of different mRNAs in *D. melanogaster* ^43^. Given this discrepancy between the efficiency and specificity upon activation of the siRNA and miRNA pathways, the finding that vertebrates have lost the siRNA-class AGOs, and therefore most likely the entire antiviral siRNA-pathway, is of particular interest for the usage of RNAi as a loss-of-function tool. In general, utilizing the RNAi-technology as a loss-of-function tool in vertebrates is much less user-friendly than in insects and nematodes^44^. For instance, when long dsRNA, and even siRNAs, is introduced in mammalian cells, other immune pathways are activated, such as the interferon and JAK-STAT pathways, which leads to a non-specific translation stop in the cell^45^. Furthermore, several species of insects and nematodes possess the remarkable ability to take up dsRNA from the environment and further spread the RNAi-signal to the rest of the body^5, 46^. Therefore, a single injection or feeding event with long dsRNA can induce potent gene silencing effects in these animals^47, 48^. Yet, the body does not take up smaller dsRNA-fragments, such as miRNAs, efficiently. In accordance with the absence of RNAi-based antiviral immunity, mammals lack efficient dsRNA-uptake mechanisms. For this reason, delivery of the RNAi signal in vertebrates requires liposome- and virus-based approaches to transport the siRNAs into the cells and tissues. Therefore, the absence of siRNA-based immunity in vertebrates might help explain the relatively insensitive and unspecific RNAi-response observed in most vertebrates.

In conclusion, we describe for the first time that there are three conserved Argonaute lineages in animals and that vertebrates lost the antiviral AGO Argonaute lineage.

## Methods

### Phylogenetic analyses

Transcript sequence information for Argonaute proteins of different chordate, insect, chelicerate, nematode, flatworm, cnidarian and poriferan species were retrieved from NCBI, flybase and wormbase. Detailed information on the used sequences is available in the Supplementary Table S1. Shortly, regarding the chordates, Argonaute sequence information of *Homo sapiens* (primates), *Alligator mississippiensis* (Crocodilla), *Gallus gallus* (Galliformes), *Danio rerio* (Cypriniformes), *Branchiostoma floridae* (Amphioxiformes) was used. The selected nematode species for sequence analyses were *C. elegans* (Rhabditidae), *Ascaris suum* (Ascaridia), *Brugia malayi* (Spiurida) and *Necator americanus* (Strongylida). Concerning the Arthropoda, *D. melanogaster* (Diptera), *Bombyx mori* (Lepidoptera), *Tribolium castaneum* (Coleoptera), *Schistocerca gregaria* (Orthoptera), *Parasteatoda tepidariorum* (Araneae), *Ixodes scapularis* (Ixodida) and *Limulus polyphemus* (Xiphosura) were selected for phylogenetic analyses. The flatworm species under investigation are *Echinococcus multilocularis* (Cestoda), S*chistosoma japonicum* (Trematoda) and *Schmidtea mediterranea* (Rhabditophora). For the Cnidaria, Argonaute sequences of *Acropora digitifera* (Acroporidae), *Acropora millepora* (Acroporidae), *Orbicella faveolata* (Merulinidae) and *Nemastella vectensis* (Actiniaria) were used. Finally, regarding the Porifera, Argonautes were identified in the available genome database of the sponge *Amphimedon queenslandica* (Haplosclerida) and in the available sequence data of the sponges *Ephydatia fluviatilis* (Haplosclerida) and *Oscarella lobularis* (Homosclerophorida). The deduced amino acid sequences, determined by *in silico* translation using Prosite (ExPASy, ETH Zurich), was used to predict the PIWI protein domains (Pfam, Sanger institute). The amino acid sequences of the PIWI domains were aligned with T-coffee software using the PAM matrix (EMBL-EBI). The region with highest sequence homology, which was approximately 200 amino acids in length, was selected using Jalview software. The latter was used for the construction of a phylogentic tree using the maximum likelihood (ML) method with 100 bootstraps (PhylML). The Jones-Taylor-Thornton (JTT) substitutions model was used, since we determined with MEGA7 that this is the best substitution model for our dataset. The final tree was visualized in MEGA7. All Argonaute proteins found in the studied animal species were used, with the exception of several SAGO proteins of *C. elegans*. This nematode encodes 19 different SAGO proteins. Previous studies have shown that all *C. elegans* SAGO proteins cluster in one clade and overrepresentation of one branch can however istort the formation of the correct phylogenetic tree. Therefore, six *C. elegans* SAGO orthologues were randomly selected for analyses. On the other hand, all SAGO proteins found in the other nematode species under investigation were included in the data set.

### Rate of molecular evolution analyses

Codon-based multiple sequence alignments were performed in MEGA7, using the nucleic acid sequence of the entire coding region of *argonautes*. ML-phylogentic trees were constructed in MEGA7 to confirm the correct identity of the selected Argonaute sequences. The amount of nonsynonymous (Ka) versus synonynomous (Ks) nucleic acid substitutions was estimated by means of pairwise comparisons (runmode = −2) in Codeml (implemented in the PAML package) and by ML-tree-based methods using KaKs Calculator (UiB-CBU). The *Drosophila* species (*sp*.) used in these analyses were *D. melanogaster*, *D. simulans*, *D. sechellia*, *D. yakuba* and *D. erecta*, retrieved from flybase. The primate species included in the analyses were *H. sapiens* (human), *Pan troglodytes* (chimpanzee), *Pan paniscus* (bonobo), *Gorilla gorilla* (western gorilla), *Pongo abelli* (Bornean orangutan), *Macaca mulatta* (rhesus macaque) and *Callithrix jacchus* (marmoset). Regarding the *Caenorhabditis* s, we retrieved *argonaute* sequence information of *C. elegans*, *C. briggsae*, *C. remanei* and *C. brenneri*. Besides determining the Ka/Ks values using homologues sequences of closely related species, we also determined the rate of molecular evolution upon comparison of homologous sequences of more distantly related species. Due to high sequence divergence (especially between the siRNA-class AGO and PIWI proteins), only the tree-based method (KaKs calculator, CBU) was used for Ka/Ks calculations. We used sequences of insect species of different insect orders, namely of *D. melanogaster* (Diptera), *B. mori* (Lepidoptera), *T. castaneum* (Coleoptera) and *S. gregaria* (Orthoptera). For the vertebrates, we used sequence information of *H. sapiens* (Primates), *Mus musculus* (Rodentia), *A. mississippiensis* (Crocodilla), *G. gallus* (Galliformes) and *D. rerio* (Cypriniformes). Finally, the nematode species selected for this analysis were *C. elegans* (Rhabditidae), *A. suum* (Ascaridia*), B. malayi* (Spiurida) and *N. americanus* (Strongylida). The number of data points used for pairwise and tree-based comparison of *argonaute* sequences within the *Drosophila* genus are n = 10 and n = 8, respectively (Fig. 2A and B). For the tree-based comparison of *argonaute* sequences of different insect orders n = 6 data points were used in the dataset. The *ago4* sequence of *G. gorilla* was not included in the dataset, due to the presence of a single insertion that disturbed correct alignments. The number of data points used for the estimation of Ka/Ks values of primate *argonautes* was n = 16 for pairwise and n = 12 for tree-based comparison of *ago4* sequence, and n = 21 and n = 14 for pairwise and tree-based comparison of the other primate *argonaute* sequences. Finally, the Ka/Ks values that were estimated by means of tree-based comparison generated 6 different data points (n = 6) for the *Caenorhabditis* and nematode data sets. Significant differences between Ka/Ks values of the *argonaute* sequences and the housekeeping genes *glyceraldehyde 3-phosphate dehydrogenase* and *apha-tubulin1a* was determined with the MWU-test.

### Site-specific selection analyses

Codeml provides several models that allow the Ka/Ks ratio to vary among sites, which were used to test for codon-specific directional selection. For this purpose, control codon based phylogenetic ML-tree reconstructions were made with MEGA7 in combination with the codon-based nucleic acid sequence alignments used for global Ka/Ks estimations. Comparisons of model 7 (Neutral, beta) and model 8 (Positive Selection, beta and Ka/Ks > 1) were used to test for signs of positive selection. Significant difference between these models was determined in R using the Chi-Square Distribution test (df = 2) for the Likelihood ratio test (LRT) values.

### Data Availability

All sequence information is publicly available on Genbank, flybase and wormbase. The accession numbers of all Argonaute sequences used for the construction of the phylogenetic tree our summarized in Supplementary Table S1.

## Electronic supplementary material

Supplementary information
