# Supplementary material for: The evolution of animal Argonautes: evidence for the absence of antiviral AGO Argonautes in vertebrates
Source: Sci Rep. 2017 Aug 23;7:9230. doi: 10.1038/s41598-017-08043-5 (PMC5569025; doi:10.1038/s41598-017-08043-5)
Supplement: Supplementary file 1 — Supplementary information [file 41598_2017_8043_MOESM1_ESM.pdf]

The evolution of animal Argonautes: evidence for the absence of antiviral AGO Argonautes in vertebrates

Niels Wynant*, Dulce Santos and Jozef Vanden Broeck

Molecular Developmental Physiology and Signal Transduction, KU Leuven, Naamsestraat 59, P.O. Box 02465, B-3000 Leuven, Belgium.

* Corresponding author: E-mail address: Niels.Wynant@kuleuven.be

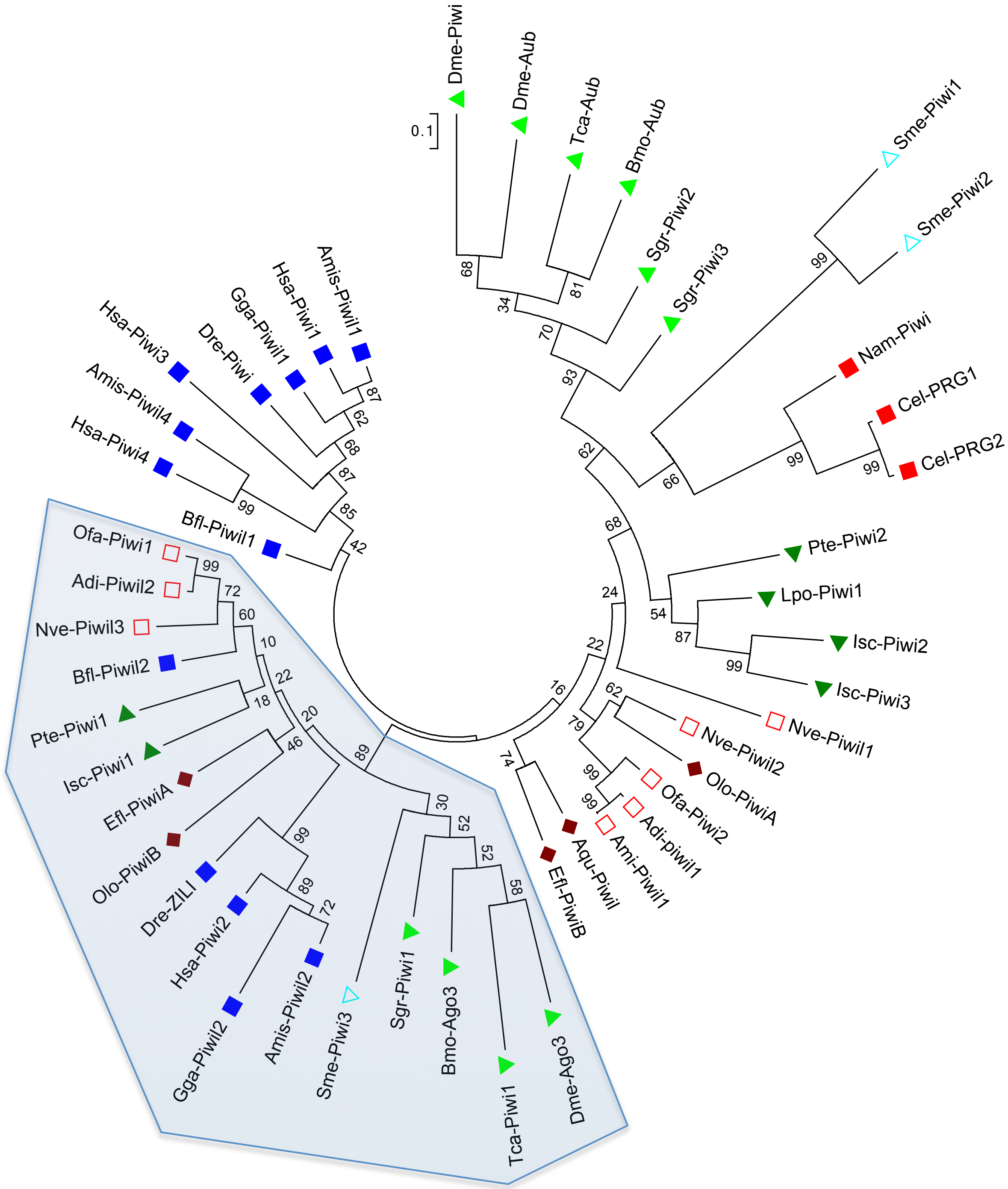

Supplementary Figure S1, related to Figure 1

Description	Max score	Total score	Query cover	E value	Identity	Accession n°	Ago-type
Bf-AGO2; Branchiostoma floridae hypothetical protein. mRNA	388	388	61%	1.00E-115	35%	XM_002589997.1	miRNA-class AGO

Supplementary Table S1, related to Figure 1

Phylum	class	order	species	gene name	Accession number	Argonaute-type*
Chordata	Mammalia	Primates	*Homo sapiens*	Hsa-Ago1	NM_012199.4	miRNA-class AGO
				Hsa-Ago2	XM_011516968.2	miRNA-class AGO
				Hsa-Ago3	NM_177422.2	miRNA-class AGO
				Hsa-Ago4	XM_011540884.2	miRNA-class AGO
				Hsa-piwil1	XM_017020229.1	PIWI
				Hsa-piwil2	NM_001135721.1	PIWI
				Hsa-piwil3	NM_001008496.3	PIWI
				Hsa-piwil4	NM_152431.2	PIWI
	Reptila	Crocodilla	*Alligator mississippiensis*	Amis-Ago1	XM_006269876.3	miRNA-class AGO
				Amis-Ago2	XM_006258700.3	miRNA-class AGO
				Aism-Ago3	XR_002090536.1	miRNA-class AGO
				Aism-Ago4	XM_006269875.3	miRNA-class AGO
				Amis-Piwil1	XM_019486674.1	PIWI
				Amis-Piwil2	XM_019491533.1	PIWI
				Amis-Piwil4	XM_006018646.1	PIWI
	Aves	Galliformes	*Gallus gallus*	Gga-Ago1	NM_001317212.1	miRNA-class AGO
				Gga-Ago3	NM_001030900.1	miRNA-class AGO
				Gga-Ago4	NM_001039276.1	miRNA-class AGO
				Gag-Piwil1	XM_015275263.1	PIWI
				Gga-Piwil2	XM_015273687.1	PIWI
	Actinopterygii	Cypriniformes	*Danio rerio*	Dre-Ago1	XM_005158472.3	miRNA-class AGO
				Dre-Ago2	XM_009294368.2	miRNA-class AGO
				Dre-Ago3	XM_005159456.2	miRNA-class AGO
				Dre-Ago4	NM_001252559.2	miRNA-class AGO
				Dre-ZILI	FJ168029.1	PIWI
				Dre-PIWIL1	NM_183338.1	PIWI
	Leptocardii	Amphioxiformes	*Branchiostoma floridae*	Bfl-AGO1	XM_002589344.1	miRNA-class AGO
				Bfl-AGO2	XM_002589997.1	miRNA-class AGO
				Bfl-Piwil1	KC516709.1	PIWI
				Bfl-Piwil2	KC516710.1	PIWI
Nematoda	Chromadorea	Rhabditidae	*Caenorhabditis elegans*	Cel-Alg1	NM_001270310.1	miRNA-class AGO
				Cel-Alg2	NM_061436.3	miRNA-class AGO
				Cel-Ergo1	NM_070961.7	siRNA-class AGO
				Cel-Rde1	NM_171525.4	siRNA-class AGO
				Cel-SAGO1	NM_072209.3	WAGO
				Cel-SAGO2	NM_182059.3	WAGO
				Cel-WAGO1	NM_059644.5	WAGO
				Cel-WAGO2	F55A12.1	WAGO
				Cel-PPW2	NM_059134.5	WAGO
				Cel-CSR1	NM_001047473.3	WAGO
				Cel-Hpo24	NM_060242.4	Hpo24-like*
				Cel-Alg3/4	NM_066791.4	alg3/4-like*
				Cel-PRG1	NM_059720.5	PIWI
				Cel-PRG2	NM_068593.6	Piwi
		Ascaridia	*Ascaris suum*	Asu-Alg1	HQ611964.1	miRNA-class AGO
				Asu-ALG7	HQ611968.1	siRNA-class AGO
				Asu-ALG5	HQ611966.1	siRNA-class AGO
				Asu-ALG6	HQ611967.1	Hpo-like*
				Asu-ALG4	HQ611965.1	alg3-like*
				Asu-WAGO	HQ611970.1	WAGO
				Asu-WAGO2	HQ611971.1	WAGO
				Asu-WAGO3	HQ611972.1	WAGO
				Asu-CSR1	HQ611969.1	WAGO
				Asu-NRDE3	HQ611973.1	WAGO
	Secernentea	Spiurida	*Brugia malayi*	Bma-Alg1	XM_001894221.1	miRNA-class AGO
				Bma-Alg2	XM_001894221.1	miRNA-class AGO
				Bma-Rde1	XM_001898588.1	siRNA-class AGO
				Bma-Alg3	XM_001891898.1	alg3/4-like*
				Bma-NRDE3	XM_001900278.1	WAGO
				Bma-WAGO2	XM_001895162.1	WAGO
				Bma-WAGO1	XM_001895162.1	WAGO
				Bma-CSR1	XM_001901544.1	WAGO
		Strongylida	*Necator americanus*	Nam-ALG1	XM_013443759.1	miRNA-class AGO
				Nam-RDE1-like	XM_013450933.1	siRNA-class AGO
				Nma-WAGO1	XM_013451313.1	WAGO
				Nam-WAGO2	XM_013437700.1	WAGO
				Nam-PIWI	XM_013437264.1	PIWI

Phylum	class	order	species	gene name	Accession number	Argonaute-type*
Arthropoda	Insecta	Diptera	*Drosophila melanogaster*	Dme-Ago1	NM_079010.4	miRNA-class AGO
				Dme-Ago2	NM_140518.3	siRNA-class AGO
				Dme-Ago3	NM_001043162.3	PIWI
				Dem-Piwi	AF104354.1	PIWI
				Dme-Aub	NM_057386.5	PIWI
		Lepidoptera	*Bombyx mori*	Bmo-Ago1	NM_001102461.1	miRNA-class AGO
				Bmo-Ago2	XM_012693089.1	siRNA-class AGO
				Bmo-Ago3	XM_012695095.1	PIWI
				Bmo-Aub	XM_012689822.1	PIWI
		Coleoptera	*Tribolium castaneum*	Tca-Ago1	XM_015982502.1	miRNA-class AGO
				Tca-Ago2a	M_008202051.2	siRNA-class AGO
				Tca-Agob	XM_008194763.2	siRNA-class AGO
				Tca-Piwi1	XM_008194203.2	PIWI
				Tca-Aub	XM_008198081.2	PIWI
		Orthoptera	*Schistocerca gregaria*	Sgr-Ago1	KY555621	miRNA-class AGO
				Sgr-Ago2	JX516788.1	siRNA-class AGO
				Sgr-Piwi1	KY555624	PIWI
				Sgr-Piwi2	KY555622	PIWI
				Sgr-piwi3	KY555623	PIWI
	Arachinda	Ixodida	*Ixodes scapularis*	Isc-Ago1	XM_002413143.1	miRNA-class AGO
				Isc-Ago2	XM_002412215.1	siRNA-class AGO
				Isc-Ago3	XM_002412954.1	miRNA-class AGO
				Isc-Ago4	XM_002405620.1	siRNA-class AGO
				Isc-Ago5	XM_002399349.1	miRNA-class AGO
				Isc-Ago5	XM_002401346.1	PIWI
				Isc-Piwi1	XM_002413940.1	PIWI
				Isc-Piwi2	XM_002411725.1	PIWI
				Isc-Piwi3	XM_002399349.1	PIWI
	Arachnida	Araneae	*Parasteatoda tepidariorum*	Pte-Ago1	XM_016050247.1	miRNA-class AGO
				Pte-Ago2	XM_016050249.1	siRNA-class AGO
				Pte-Ago3	XM_016074512.1	miRNA-class AGO
				Pte-Ago4	XM_016050252.1	siRNA-class AGO
				Pte-Piwi1	XM_016048783.1	PIWI
				Pte-Piwi2	XM_016075529.1	PIWI
	Merostomata	Xiphosura	*Limulus polyphemus*	Lpo-Ago1	XM_013931086.1	miRNA-class AGO
				Lpo-Ago2	XM_013926285.1	siRNA-class AGO
				Lpo-Piwi1	XM_013918768.1	PIWI
Plathyelminthes	Cestoda	Cyclophyllidea	*Echinococcus multilocularis*	Emu-Ago1	FN690741.1	miRNA-class AGO
				Emu-Ago2	KF768022.1	siRNA-class AGO
	Trematoda	Stringeidida	*Schistosoma japonicum*	Sja-Ago1	KC345759.1	miRNA-class AGO
				Sja-Ago2	KC345760.1	siRNA-class AGO
				Sja-Ago3	KC345761.1	siRNA-class AGO
	Rhabditophora	Tricladida	*Schmidtea mediterranea*	Sme-Ago1	JF263459	miRNA-class AGO
				Sme-Piwi1	DQ186985.1	siRNA-class AGO
				Sme-Piwi2	DQ186986.1	PIWI
Cnidaria	Anthozoa	Scleractinia	*Acropora digitifera*	Adi-Ago1	XM_015896058.1	miRNA-class AGO
				Adi-Ago2	XM_015896084.1	siRNA-class AGO
				Adi-Ago3	XM_015896077.1	siRNA-class AGO
				Adi-Ago4	XM_015896088.1	siRNA-class AGO
				Adi-Piwil1	XM_015921219.1	PIWI
				Adi-Piwil2	XM_015908676.1	PIWI
			Acropora millipora	Ami-Ago1	JR981312.1	miRNA-class AGO
				Ami-Ago2	JT022739.1	siRNA-class AGO
				Ami-Piwil1	EZ001072.1	PIWI
				Ami-Piwil2	EZ012267.1	PIWI
			Orbicella faveolata	Ofa-Ago1	XM_020761752.1	siRNA-class AGO
				Ofa-Ago2	XM_020761847.1	siRNA-class AGO
				Ofa-Ago3	XM_020761831.1	siRNA-class AGO
				Ofa-Piwi1	XM_020745504.1	PIWI
				Ofa-Piwi2	XM_020776165.1	PIWI
		Actiniaria	*Nemastella vectensis*	Nve-Ago1	KF192061.1	miRNA-class AGO
				Nve-Ago2	KF192062.1	siRNA-class AGO
				Nve-Piwil	KU746924.1	PIWI
				Nve-Piwil	XM_001641944.1	PIWI
				Nve-Piwil	XM_001626077.1	PIWI
Porifera	Demospongiae	Haplosclerida	*Amphimedon queenslandica*	Aqu-Ago1	XM_019995499.1	miRNA-class AGO
				Aqu-Ago2	XM_020008918.1	siRNA-class AGO
				Aqu-Piwil	XM_020007102.1	PIWI
			Ephydatia fluviatilis	Efl-Ago	AB533507	miRNA-class AGO
				Efl-PiwiA	XM_020761752	PIWI
				Efl-PiwiB	XM_020761753	PIWI
			Oscarella lobularis	Olo-Ago	KX216818.1	/
				Olo-PiwiA	KX216816.1	PIWI
				Olo-PiwiB	KX216817.1	PIWI
Bacteroidetes	Bacteroidetes	Bacteroidales	*Bacteroides fragilis*	OUT-GROUP	FQ312004.1	/
Firmicutes	Bacilli	Bacillales	*Exiguobacterium sp.*	OUT-GROUP	CP001615.1	/

Supplementary Table S2, related to Figure 1.

<u>Legends:</u>

Figure S1: Unrooted maximum likelihood phylogenetic tree (100 bootstraps) using the AGO Argonaute members belonging to Porifera (sponges; ◇), Cnidaria (jellyfish, coral animals and anemones, □), Chordata (■), Nematoda (◆), Arthropoda (Insecta: ▲ and Chelicerata: ▼) and Platyhelminthes (△).

Table S1: *Branchiostoma floridae* Bf-AGO2 is the chordate sequence with highest sequence similarity (depicted by lowest E-value) to *Drosophila melanogaster* Dm-AGO2.

Table S2: Detailed sequence information of the Argonaute sequences used for the construction of a Maximum Likelihood (ML-) phylogenetic tree.
